# *Nannodromus reveilleti* (Acari, Anystida, Saxidromidae) a new genus and species from South Africa

**DOI:** 10.3897/zookeys.378.6753

**Published:** 2014-02-06

**Authors:** Nestor Fernandez, Yves Coineau, Pieter Theron, Louwrens Tiedt

**Affiliations:** 1 National Council of Scientific and Technological Research (C.O.N.I.C.E.T). La Rioja University Campus. Research and Technology City. Av. Luis Mansueto de la Fuente S/N. (5300) La Rioja, Argentina; 2 Professor Emeritus in Muséum National d’Histoire Naturelle, Paris France; 3 Research Unit for Environmental Sciences and Management, North-West University, Potchefstroom Campus, 2520, South Africa; 4 Laboratory for Electron Microscopy, North-West University, Potchefstroom Campus, Private Bag X6001, Potchefstroom, 2520 South Africa

**Keywords:** Acari, Anystides, Saxidromidae, *Nannodromus* gen. n., *Nannodromus reveilleti* sp. n., South Africa, males–females

## Abstract

The description of a new genus *Nannodromus* and a new species *Nannodromus reveilleti* (Acari: Anystides: Saxidromidae) from South Africa, based on adult males and females.

## Introduction

Since 1974 studies of behavioural patterns, spermatophore types and characteristics, as well as mating and secondary characteristics of males have led to several publications (Coineau 1974, 1976; Alberti et al. 2000; Coineau et al. 2006, Alberti et al. 2010), as well as a short documentary film (Coineau and Kovoor 1982).

Intensive sampling around South Africa and neighboring countries over a period of many years, (by Y. Coineau and P.D. Theron) has led to the acquisition of many specimens. Coineau and colleagues described two new genera, namely *Bovidromus* Coineau et al. 2006 and *Rhinodromus* Coineau et al. 2006.

Obtaining large amounts of material made it possible to study some of this material of both sexes with Light Microscopy (LM) and Scanning Electron Microscopy (SEM). This and future publications aim to facilitate understanding of morphological variations, presence or absence of males, and geographical distribution of species of this very unique mite family.

According to the present study the new genus *Nannodromus* displays well definable differences as well as shared characteristics with the other three known genera of the family.

## Materials and methods

Specimens studied by means of light microscopy (LM) were macerated in lactic acid, and observed in the same medium using the open-mount technique (cavity slide and cover slip) as described by Grandjean (1949) and Krantz and Walter (2009). Drawings were made using a Zeiss-Axioscope compound microscope equipped with a drawing tube.

Measurements taken: total length (tip of rostrum to posterior edge of notogaster); width (widest part of notogaster) in micrometers (μm).

Leg chaetotaxy studies done by use of standard, polarized and phase contrast microscopes.

Some specimens were studied by means of a Scanning Electron Microscope (SEM). For this purpose, specimens preserved in ethanol were carefully rinsed by sucking them several times into a Pasteur pipette, and then transferring them to buffered glutaraldehyde (2.5%) in Sörensen phosphate buffer: pH 7.4; 0.1 m for 2 hours. After postfixation for 2 hours in buffered 2% OsO4 solution and rinsing in buffer solution, all specimens were dehydrated in a series of graded ethanols and dried in a critical point apparatus. Specimens were mounted on Al-stubs with double-sided sticky tape and then gold coated in a sputter apparatus (Alberti and Fernandez 1988; Alberti and Fernandez 1990a, 1990b; Alberti et al. 1991; Fernandez et al. 1991; Alberti et al. 1997; Alberti et al. 2007). For some studies, specimens were dissected and monitored during the lactic acid maceration process (in warm 70% lactic acid) before being stained with chlorazol black E (Coineau 1974). Measurements taken: total length (tip of rostrum to posterior edge of notogaster) and width (widest part of notogaster) in micrometres (μm). Leg chaetotaxy studies done using standard, polarized and phase contrast microscopes.

### Morphological terminology

Morphological terms and abbreviations used are those developed by Coineau 1974, 1976; Coineau and Naudo 1986 and Coineau et al. 2006.

## New taxa description

### Nannodromus gen. n.

http://zoobank.org/96115256-59DE-4C08-A075-D67C0A06BF4C

http://species-id.net/wiki/Nannodromus

#### Etymology.

The generic prefix “*nanno*” derives from “*nannos*” (Greek = dwarf English = nain French) on account of the small size of this species.

#### Diagnosis.

**Adult. Male.** Small animal of around 600 µm; male with two clearly discernible dorsal sclerites (***D*** and ***P***). Transversal furrow separating sclerites, situated at level of leg pair IV. ***D*** with naso globular shape and reticulate surface; trichobothrium simple. Dorsal paired processes. In dorsal view: digitiform; in lateral view: sabot-like (= like a wooden shoe or clog), apical zone rounded, arching upwards; in frontal view: cylindric with a blunt hornlike structure, directed paraxially, apical part curving upwards. Small setae *pa* situated antiaxially of dorsal paired processes, provided with very small asperities. Towards anterior, behind dorsal digitiform paired processes, in saggital position, conspicuous U-shaped depression. Anterior and posterior eye, *po*, *a_1_*, *a_2_*, *b_1_* and *b_2_* setae; six depressed areas, one unpaired situated in U shaped depression and five others, paired, in depressed area; lyrifissures *ly*, *ia*.

Sclerite ***P*** complete, with three pairs of depressed areas; setae *c_1_*, *c_2_*, *d_1_*, *d_2_*, *e_1_*, *e_2_*, *e_3_*; lyrifissure *im* and *ip*. Chelicera exhibiting neotrichy (16–20 setae).

#### Type species.

*Nannodromus reveilleti* gen. n., sp. n.

### Nannodromus reveilleti gen. n., sp. n.

http://zoobank.org/4582C9B7-96EF-4D55-8D78-9EEA69C15A45

http://species-id.net/wiki/Nannodromus_reveilleti

#### Etymology.

The species is dedicated in homage to the late Pierre Reveillet, Pharmacist, Biologist and Entomologist from Valence, France; an intimate friend and assiduous and tireless contributor to many missions in Africa.

#### Type material.

**Holotype** male and two female paratypes, N’Wanetsi, Kruger National Park, South Africa: 24°27'30.56"S, 31°58'35.30"E altitude 171 m. This area is bordered by the South African provinces Limpopo and Mpumalanga, and it also shares a border with Mozambique.

Basic volcanic rocks (tholeiites, picrite basalts and nephelinites), vegetation type Lowveld Savanna.

Material was collected by Y. Coineau, R. Cléva, P. Reveillet coll. 02 February 1996; Y. Coineau and P. Theron coll. 12 February 2001 (N’Wanetsi) and P.Theron 2010 and 12 February 2012 (N’Wanetsi) deposited in the Collection of the Muséum National d’Histoire Naturelle, Paris, France, preserved in 70% ethanol; two **paratypes** (1 male/1 female) deposited in Museum d’Histoire Naturelle, Geneva, Switzerland, preserved in 70% ethanol.

Type locality: Kruger National Park, N’Wanetsi: 24°27'30.56"S, 31°58'35.30"E altitude 171 m.

#### Diagnosis.

**Males.** shape: Elongate oval; colour yellowish-light brown.

*Dorsal region*. Sclerite ***D*:** polygon network microsculpture, from sagittal zone towards posterior; *bp*, *oc*, *a_1_*, *a_2_*, *b_1_*, *b_2_* setae. Lyrifissure *ly* situated near *op* and *ia* behind *a_2_*; cuticular stria surrounding lyrifissures on ovoid sclerite.

Basal zone of U-shaped depression harboring depressed area numbered 1. Depressed area 2 behind the bothridium. Antiaxially and behind *po*, paired anterior eyes, with convex cornea and ovoid, slightly concave posterior eye. Angle between *oa* and *op* 90 degrees. Zone of very complex microstructure surrounding *oa* and *op*. Depressed area 3 behind 2; depressed area 4 between *a_1_*, *a_2_* setae. Depressed area 5 behind this zone, with lyrifissure *ia*. Near posterior limit of aspidosoma, setae *b_1_*, *b_2_* with depressed area 6 between them. Cheliceral setae: *cha*, simple; *chb* bifid. Other setae neotrichous. Sclerite ***P*** complete, polyhedral network microsculpture; depressed area 7 situated anteriorly near transversal furrow; depressed area 8 behind *c_2_* setae; 9 behind *d_2_* at level of *e_3_* setae.

*Ventral region*. Epimeric formula (2, 2, 3, 3); three pairs of aggenital setae; progenital lips, five pairs of setae. Along paraxial edge a line of short setae. Four pairs of anal setae; four pairs of adanal setae; five pairs of *ps* setae; lyrifissure *ih* clearly visible. All legs with apoteles, three heteromorphic claws, one pair isomorphic, another small unpaired medial hook. Paired claws with two different types of barbs one triangular with tooth-like appearance; another thin curved barb. Hypertrophic setae on leg I, tibia and tarsi; tibial claw I present.

**Females.** Shape: elongate oval, color: light yellow

*Dorsal region*. Three sclerites: anterior (***A***), middle (***M***), posterior (***P***). Striated transversal furrrow separating sclerites. ***A*** unmpaired, triangular, anterior part rectangularly shaped. Bothridium, trichobothria, anterior eye, posterior eye, setae *po*, *a_1_*, *a_2_*; depressed areas 1, 2, 3, 4 present. ***M*** paired, rectangular to ovoid, *b_1_*, *b_2_*, setae; depressed areas 5, 6 present. ***P*** variable; several possibilities from one unique unpaired sclerite or divided into series of microsclerites. First case: sclerite ***P*** unique, unpaired, with *c_1_*, *c_2_*, *d_1_*, *d_2_*, *e_1_*, *e_2_*, *e_3_* and depressed areas 7, 8, 9; lyrifissures *im*, *ip*. Second case: sclerite ***P*** divided into five paired microsclerites and one unpaired microsclerite. Paired microsclerites: one rounded, with *c_1_*, *c_2_*, setae and depressed areas 7, 8; one small ovoid microsclerite with only *d_1_* setae; one small ovoid with *d_2_* setae. Lyrifissures (*im*, *ip*) are isolated and surrounded by microsclerites. Unpaired crescent-shaped microsclerite with *e_1_*, *e_2_*, *e_3_* setae and depressed area 9. Epimeric formulae (3-3-2-3). Aggenital sclerite triangular, three pairs of setae. Progenital lips 5-7 pairs of setae; paraxial edge numerous aligned setae. Setae *ad*, *an*, and *ps* as in male. Only leg IV presenting sexual modifications with large number of hypertrophic setae and isomorphic apotele claws. Legs I, II, III, heteromorphic apotele.

#### Description.

***Measurements*:** *Males* SEM: 618 μm (615–646) × 215 (213–227) μm (material used for SEM studies not deposited).

LM: 628 μm (622–645) × 315 μm (297–322) (measurements of specimens deposited in Museum National d’Histoire Naturelle, France, and Geneva Natural History Museum, Switzerland.

*Females* SEM: 658 μm (656-664) × 301μm (296–306) (material used for SEM studies not deposited).

LM: 651 μm (643–657) × 325 μm (322–335) (measurements of specimens deposited in Museum National d’Histoire Naturelle, France, and Geneva Natural History Museum, Switzerland).

**Figure 1.**
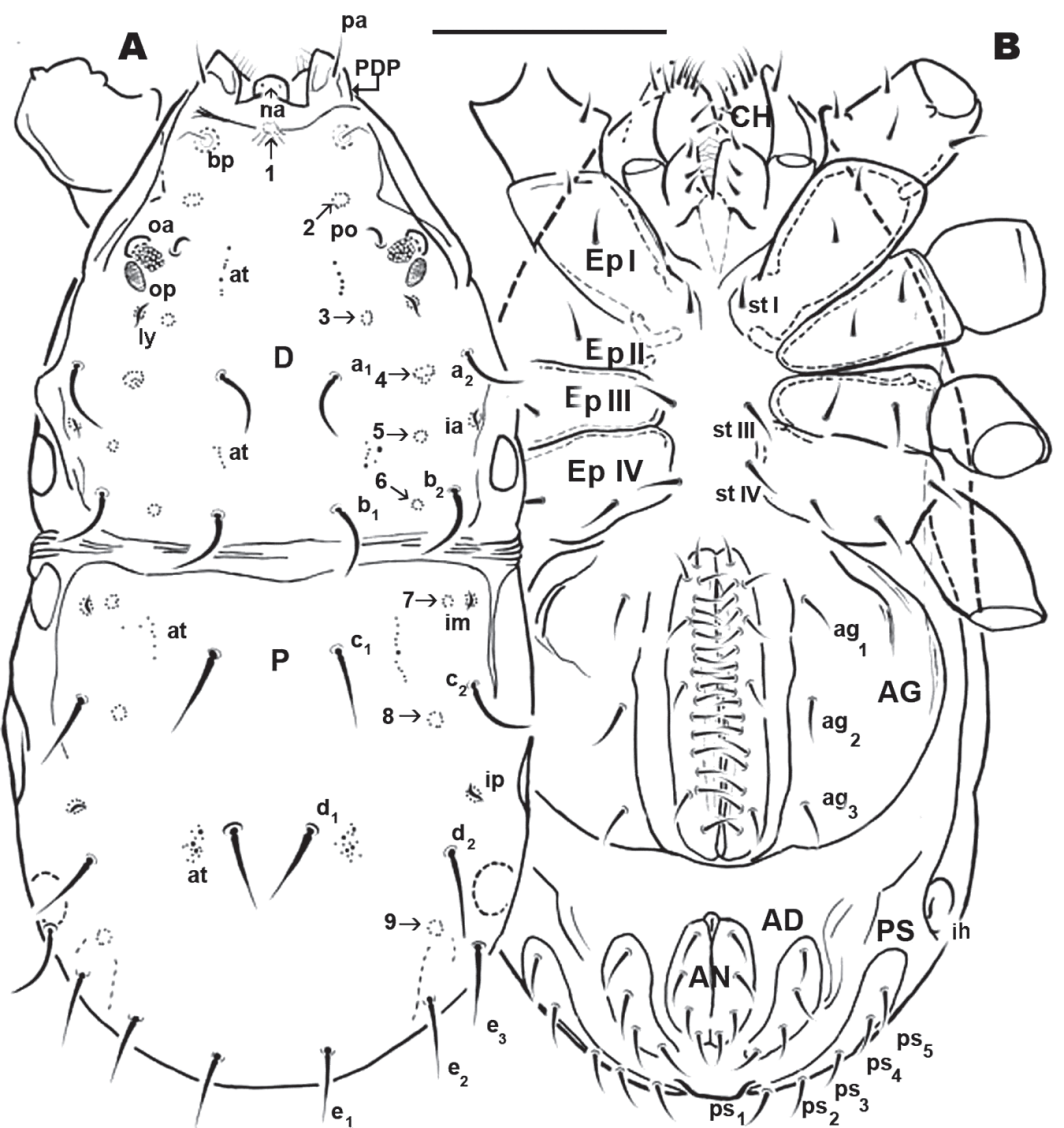
*Nannodromus reveilleti* gen. n., sp. n. Male. **A** dorsal view **B** ventral view. Scale bar: **A**, **B** = 130 μm.

**Figure 2.**
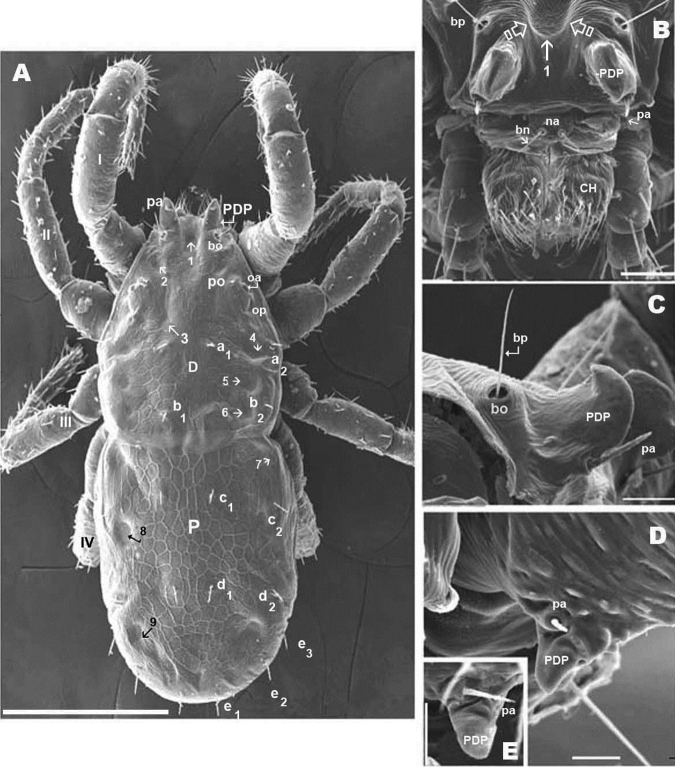
*Nannodromus reveilleti* gen. n., sp. n. Male. **A** dorsal view **B** frontal view **C** lateral view aspidosoma, anterior part **D–E** Prodorsal paired processes, details. Scale bar: **A** = 200 μm; **B** = 50 μm; **C** = 20 μm; **D** = 10 μm; **E** = 10 μm.

***Shape*:** elongate oval; **Male:** Figures 1A, B; 2A; **Female:** Figures 8A, B; 9A

***Color*:** specimens without cerotegument yellowish-light brown, slightly shiny when observed in reflected light. Males vaguely yellowish.

#### Integument.

SEM-studies assisted greatly in complementing LM observations, particularly in studies of cuticular microsculpture and gender differences.

**Males** (LM and SEM observations).

*Dorsal region*: two clearly discernible sclerites (***D***, dorsal and ***P***, posterior). Conspicuous transversal furrow, separating sclerites, situated at level of legs IV. Transversal furrow constituted by a series of fine parallel cuticular striae (Figures 1A; 2A).

**Figure 3.**
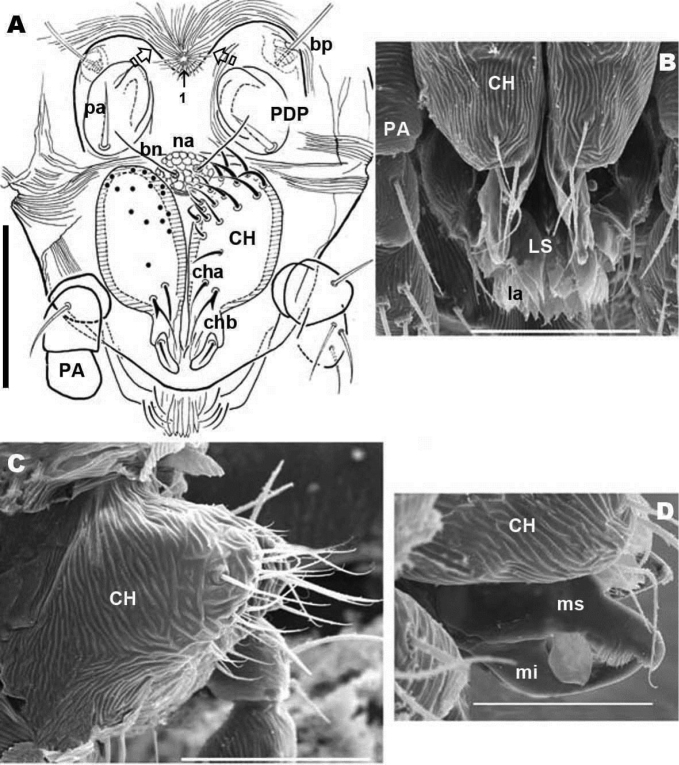
*Nannodromus reveilleti* gen. n., sp. n. **A+C** male **B+D** female **A** frontal view (LM observation) **B** frontal view (SEM observation) **C** chelicerae left (SEM observation), paraxial view **D** chelicerae (SEM observation), inferior and superior digits. Scale bar: **A**, **B**, **C** = 50 μm; **D** = 10 μm.

Sclerite ***D*** presenting a series of well-defined depressed areas, the first (Figures 1A, 2A, 3A) indicated by number 1, is unpaired, situated in U-shaped depression (indicated by white arrow) (Figures 2B; 3A), placed at level of the trichobothria and slightly behind dorsal paired processes (*PDP*) (Figures 1A, 2A; 3A); microsculpture of depressed area constituted by fine cuticular striae (Figures 3A; 8C) (in both sexes and in all cases the depressed areas display similar microsculpture). All other depressed areas on this sclerite paired: second depression (smaller than first) (Figure 1A indicated by 2 and simple arrow) between anterior eye (*oa*) and trichobothria (*bp*). Third depression (indicated by 3) situated posterior and paraxially to posterior eye (*op*). Series of three aligned depressions (4-6) of similar type between setae *a_1_* and *a_2_* to *b_1_* and *b_2_* (Figure 1A).

Conspicuous area with irregular polygon network (Figure 1A) situated in sagittal zone behind *PDP*, between both *bp*, and zone delimited by *oc* and setae *a_1_*, *a_2_*, *b_1_*, *b_2_* towards the transversal furrow.

**Figure 4.**
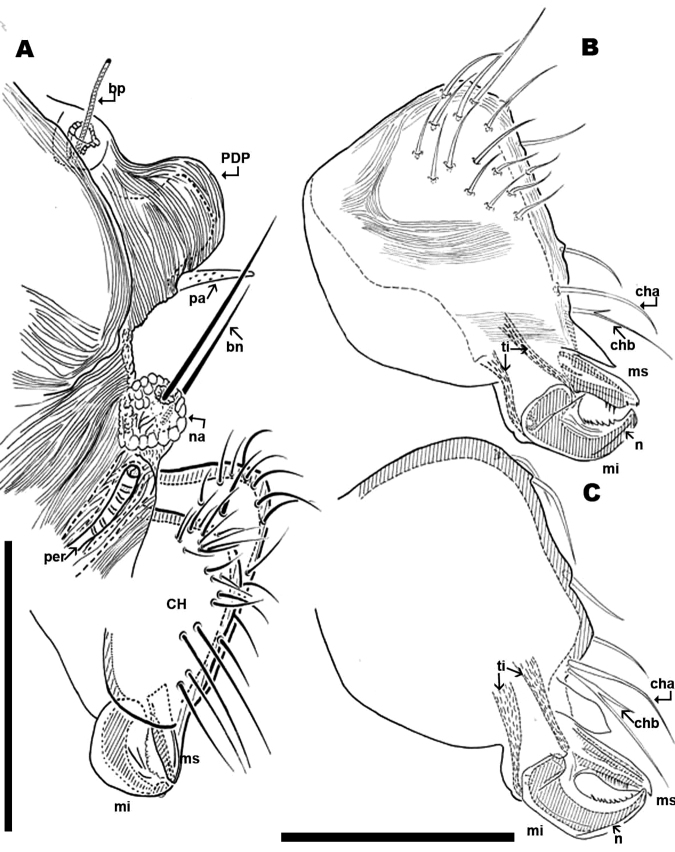
*Nannodromus reveilleti* gen. n., sp. n. **A, B** Male **C** Female **A** anterior region with antiaxial view of right chelicera **B** left chelicera, paraxial view **C** left chelicera, paraxial view. Scale bar: **A**, **B**, **C** = 100 μm.

Chelicerae (Figures 2B, 3A, 3B, 3C, 3D, 4B) and zone adjacent to *bp* at base of *PDP*, surrounding naso (*na*) and near peritremal zone (*per*) presenting very fine striated cuticular surface (Figures 2B, C, D; 3A, 4A).

**Figure 5.**
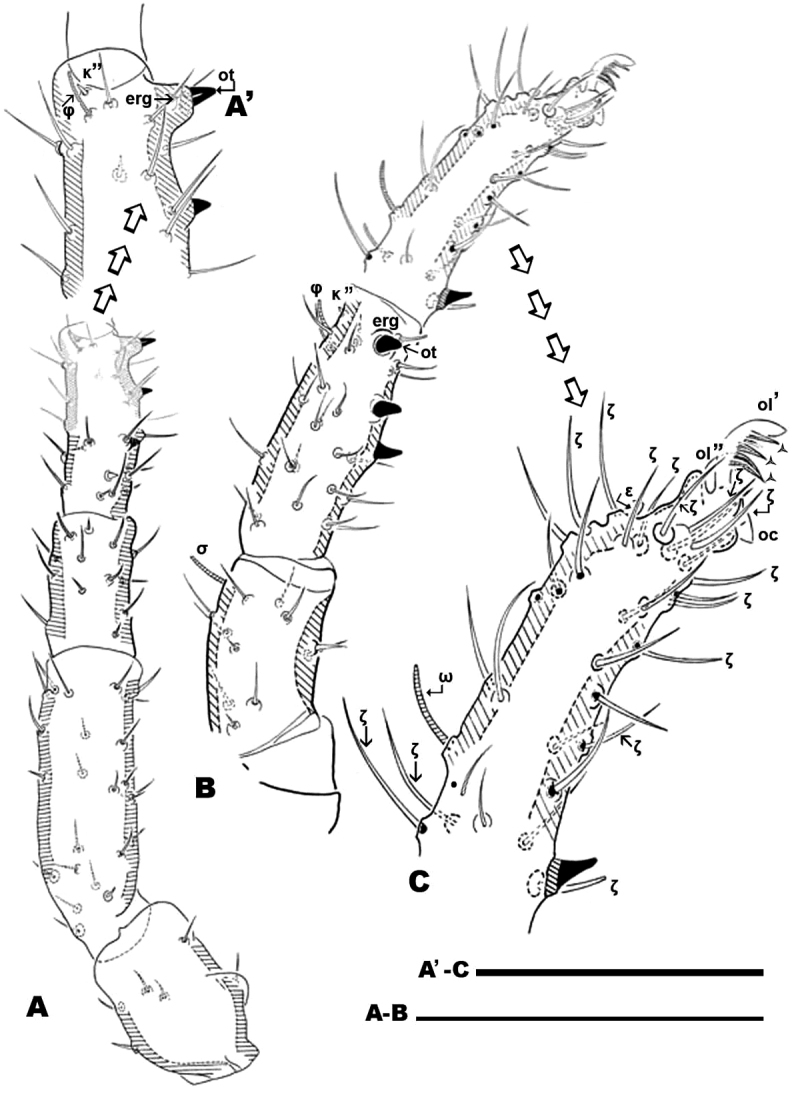
*Nannodromus reveilleti* gen. n., sp. n. Male. Leg I **A** dorsal view **B** lateral view, distal paraxial segments **C** lateral view tarsus I, paraxial. Scale bar: **A**, **B** = 100 μm; **A’**, **C’** = 50 μm.

Several tendon attachment areas (*at*) clearly distinguishable (LM) between depressed zones 2-3 and 5-6 (Figure 1A).

Two lyrifissures: *ly* situated near and behind *op*, between them and 3rd depressed area (Fig. 1A), *ia* situated behind *a_2_* setae. Finely striated cuticular striae surrounding both lyrifissures, situated on ovoid sclerite of similar type to Figures 10C, D.

Posterior sclerite ***P*** presenting conspicuous polygon network, extending to neighboring transversal furrow adjacent to posterior zone including setae *e_1_*, *e_2_*, *e_3_*.

Three conspicuous depressed zones observed on sclerite: one antiaxially situated near transversal furrow (indicated by 7), two others between *c_2_* and *d_2_* setae (indicated by 8), with third in front of *d_2_* and at level of *e_3_* seta (indicated by 9). A line of several *at* situated antiaxially to *c_1_* setae and another group of *at* situated antaxially to *d_1_* setae (Figure 1A).

**Figure 6.**
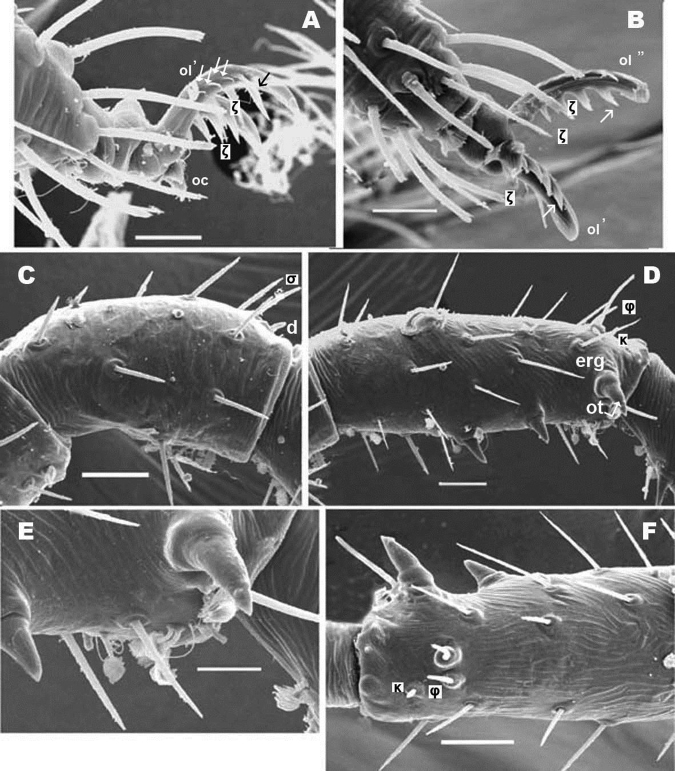
*Nannodromus reveilleti* gen. n., sp. n. Leg I. Male **A** tarsus I lateral left paraxial view **B** tarsus I dorsal view **C** genu, lateral view **D** tibia lateral left paraxial view **E** tibia, detail, paraxial left **F** tibia left dorsal view. Scale bar: **A**, **B** = 10 μm; **C** = 20 μm; **D** = 20 μm, **E** = 10 μm; **F** = 20 μm.

Legs appear smooth but in SEM, series of very fine aligned striae and polygon network visible (Figures 6C, D, E, F).

**Figure 7.**
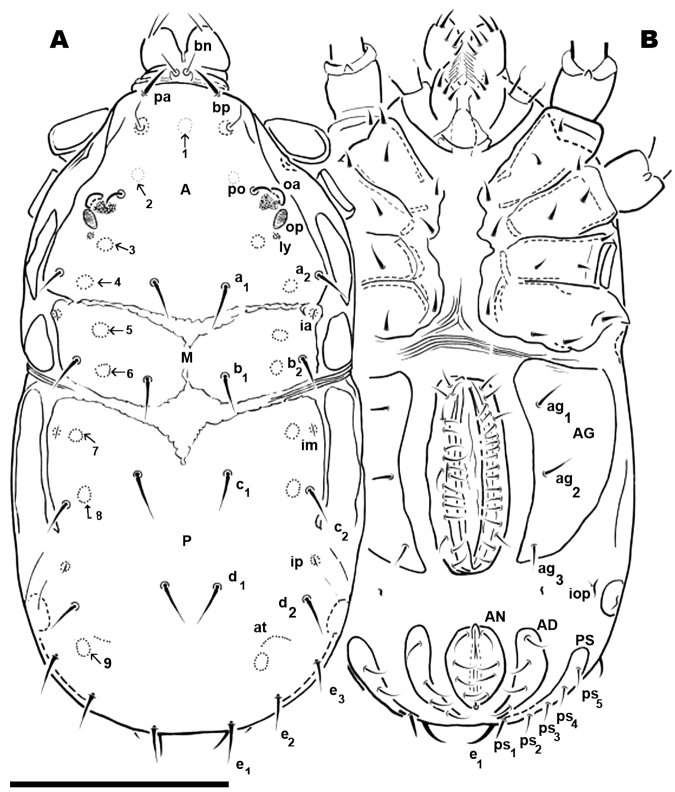
*Nannodromus reveilleti* gen. n., sp. n. Female. **A** dorsal view **B** ventral view. Scale bar: **A**, **B** = 200 μm.

**Females. LM observations** (Figure 7). Three sclerites present: ***A*, *M***, ***P. A*** with four depressed areas (1-4), one unpaired (1) situated at sagittal plane in depressed U–zone. Three pairs; the second (2) situated close to and in front of *po*; third (3) paraxial pair situated near *ly*, the last pair (4) situated between *a_2_* and *a_1_* setae (Figure 7A). Paired microsclerite ***M*** presenting lyrifissure *ia*, with two well defined paired depressed areas (5, 6), situated paraxially in front of and behind *b_2_* setal level.

Transversal furrow exhibiting finely striated cuticular microsculpture, between sclerites ***M*** and ***P*.**

Three depressed areas (7, 8, 9) on sclerite ***P***; one (7) situated anteriorly, near anterior border and close to lyrifissure *im*; second depressed area (8) paraxially close to seta *c_2_*. The third (9) situated behind *d_2_* and at level of *e_3_* setae. Lyrifissure *ip* situated between *c_2_* and *d_2_* setae. Several *at* in semi-circular line situated between setae *d_2_* and *e_3_*.

**Figure 8.**
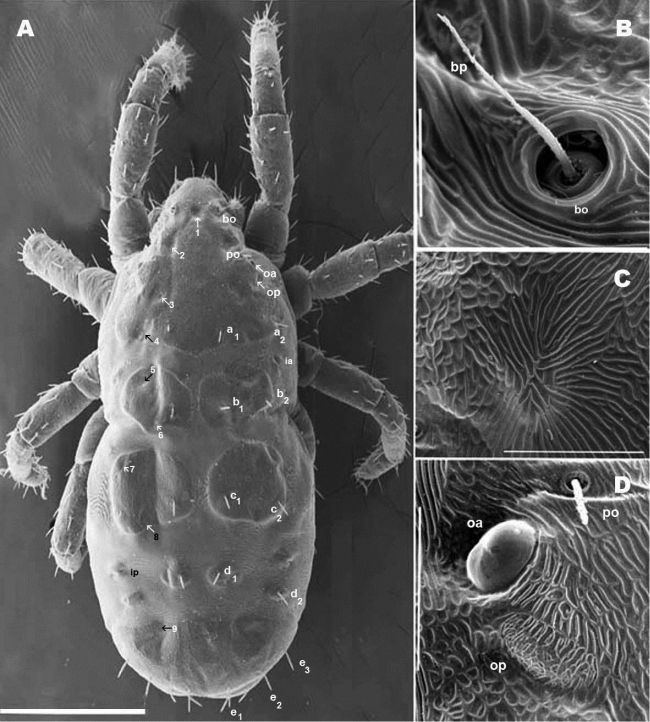
*Nannodromus reveilleti* gen. n., sp. n. Female. **A** dorsal view **B** trichobothrium **C** microsculpture depressed area **D** the two left eyes: the anterior normal with a convex cornea, the posterior regressed or modified and probably dedicated to another function. Scale bar: **A** = 200 μm; **B** = 10 μm; **C** = 20 μm; **D** = 30 μm.

**Figure 9.**
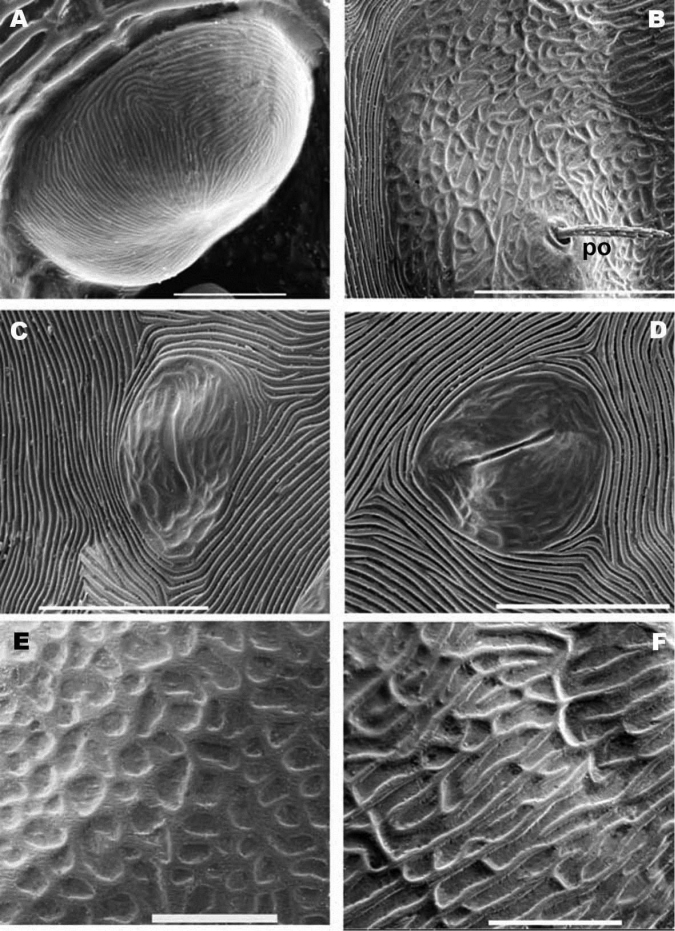
*Nannodromus reveilleti* gen. n., sp. n. Female. **A** anterior eye, cornea **B** microsculpture, zone surrounding *po* setae **C** lyrifissure, *ia* **D** lyrifissure *im* **E**, **F** microsculpture around ocular zone. Scale bar: **A** = 5 μm; **B** = 20 μm; **C**, **D** = 20 μm; **E** = 10 μm; **F** = 10 μm.

**Figure 10.**
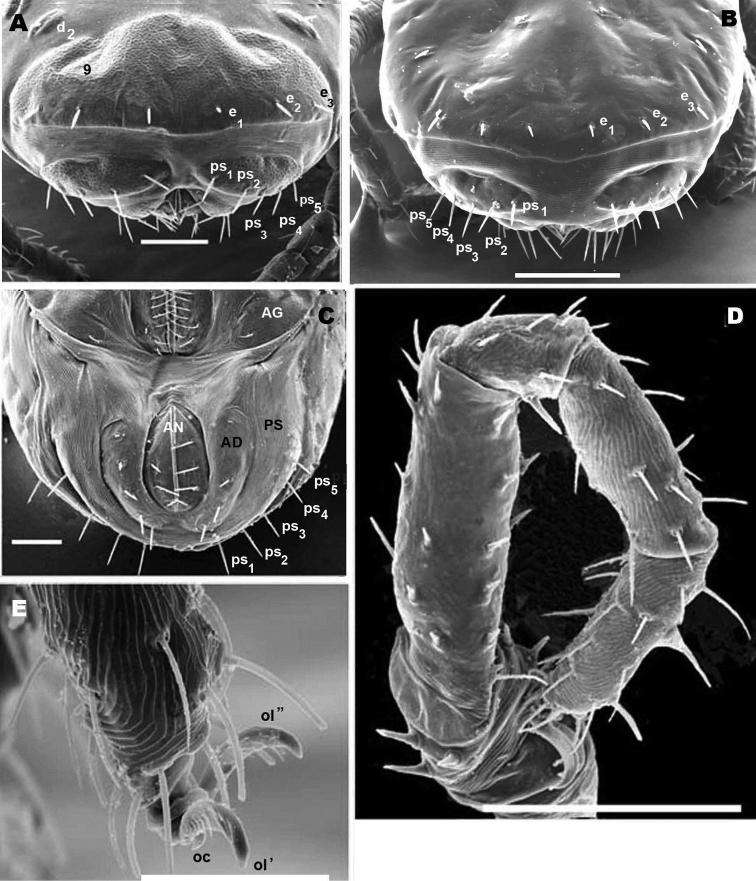
*Nannodromus reveilleti* gen. n., sp. n. Female/Male. **A** posterior view, female **B** posterior view, male **C** female, genito-anal zone **D** legs IV, female, view from above, several setae are lost **E** female, anterior zone tarsus I. Scale bar: **A** = 50 μm **B** = 70 μm; **C** = 50 μm; **D** = 100 μm; **E** = 40 μm, **F** = 10 μm.

**SEM observations** (Figures 8, 10). Very complex microsculpture of sclerite ***A*:** striated with small polygonate pattern (Figure 8B) in zone near *bp*. Area surrounding *oa* and *op*, and near *po* setae, polygonate (general view Fig. 8D), but with variable network type and cell shape (Figures 9B, E, F). Sclerite ***M*** with two depressed areas (numbered 5, 6) slightly visible; lyrifissure *ia* situated outside sclerite. Large transversal furrow with finely striated cuticular ornamentations completely separating sclerites ***A*** and ***P*.**

Sclerite ***P*** in this specimen divided into several sclerites; one anterior with lyrifissure *im* and two depressed zones (numbered 7, 8) (Figure 8A). Behind this anterior sclerite; two small ovoid-circular sclerites, at base of setae *d_1_* and *d_2_* respectively, striated cuticular ornamentations surrounding each sclerite.

Each lyrifisure *ly*, *ia*, *im* and *ip* (Figure 8A), with a small rounded sclerite, surrounded by striated cuticular pattern (Figures 9C, D).

Final sclerite, in posterior position, presenting a paired depression (numbered 9) (Figures 8A; 10A).

**Remarks.** Variation observed in the female dorsal region with LM and SEM (Figure 6, 7) caused interpretive difficulties, as the number of sclerites visible under SEM differed from LM observations.

Only sclerite ***P*** (Figure 8A) was visible in our initial LM observation and a divided sclerite in SEM observation (Figure 9B). Further observations were deemed necessary, and though a number of specimens were available for study, it was considered insufficient to address the problem. P.D. Theron re-sampled in the type locality, on the same rock, and several security measures were taken to ensure we studied the same species, that we collected adult females, and that we worked with a series of specimens of the same species. We identically reproduced previous studies in LM and SEM. Males obtained in each sample did not present deviations to characteristics as pointed out.

We considered several possibilities: 1) the existence of large intraspecific variability in the number of sclerites in zone ***P*:** sclerites may be more or less visible in animals of different ages, for this reason when using light microsocopy with recently hatched specimens, and when working with lactic acid, these structures appear faintly visible or are invisible. These slightly visible sclerites are most often observed in SEM microscopy. 2) It is possible that populations with different numbers of sclerites exist. 3) Due to the succession of generations over several months, it is possible that populations exist in which the coalescence of elementary sclerites to make up a large scutum is more or less progressed.

Figures 7A and 8A illustrate the the two extremes: one with only one sclerite ***P*** (Figure 7A), and one with several sclerites (Figure 8A), but it is necessary to indicate that we observed a comprehensive range between these two.

These issues prove the importance of using both complementary technologies (LM and SEM) as well as a large number of specimens as a unique solution to resolve this type of problem.

#### Dorsal region.

**Males.** General body aspects differ greatly between *Bovidromus roussouwi*, *Rhinodromus lootsi* and *Nannodromus reveilleti*; the last is more stylized and gives the impression of more compactly built animals.

Anteriorly the aspidosoma presents the naso (*na*), globular shaped with reticulate surface; the trichobothrium (*bn*) is simple (Figures 1A, 2B, 3A, 4A). Males exhibit important particularities in the anterior zone relating to accentuated sexual dimorphism (Figures 1A, 2A, 3A, 4A). An expanded paired process (Figure 2A) plays an important role in sexual behaviour and spermatophore transfer (Alberti et al. 2007, 2010; Coineau 1974, 1976; Coineau and Kovoor 1982; Coineau et al. 2006). Digitiform dorsal paired processes (*PDP*) (in dorsal view) (Figure 2A) appear “sabot-shaped” with rounded, upwards arching apical zone (Figure 2C), cylindrical in frontal view with a paraxially directed blunt horn (Figure 2B), apical part curving upwards (Figures 2A, C; 3A). The *PDP*, presenting setae *pa* situated basally, lateral, antiaxial with length of 20 µm (22.4-19.3) (Figures 2C, D, 3A), coated by very small asperities. Both *PDP*, are parallel, slightly diverging (Figures 2B, 3A), conspicuous U-shaped depression behind them in saggital position, harboring numbered 1 depressed area in basal zone (Figures 1A, 2B, 3A). The paraxial extremities of U-depression extending in a rounded elevation which houses the trichobothrium *bp* (Figures 2B, 3A, 4A).

Simple bothridium and *bp* with asperities (Figure 8B). Depressed area 2 behind bothridium (Figure 1A); slightly antiaxially and behind depressed area, 2 small setae *po* (±10 µm length), covered with small asperities (Figure 9B) are present. Antiaxially and behind *po*, paired eyes *oa* (anterior eye) and *op* (posterior eye) (Figures 1A, 2A, 8D, 9A) present. Paired eyes show no observable differences between sexes. The *oa* is an ovoid structure of 15 µm diameter. Small furrow (Figure 8D) surrounding convex cornea. Cornea situated in small depressed area, presenting a surface of vermiculate ornamentations (Figure 9A). Ventrally and posterior to *oa*, *op* observed as an ovoid structure, slightly concave, well delimited by a surrounding line. The *op* exhibiting a particular microsculpture (Figure 8D). The angle between *oa* and *op* is 90 degrees. The zone around *oa* and *op* exhibits very complex microsculpture (Figure 8D) as illustrated in Figure 9F. Microsculpture of zone surrounding *po* setae complex (Figure 9B, E): behind and around *op* as in Figure 9E; in this same Figure, on the anterior side, striated cuticular network extending laterally to setae *po* and in front of and behind bothridia, extending to *PDP* (Figs 2C, 4A).

Setae *a_1_*, *a_2_*, and depressed zone 4: close to the posterior limit of the aspidosoma, setae *b_1_*, *b_2_* and depressed area 6 are observed. Relative lengths of setae *po*, *a*, and *b* are: *po* < *a_1_*, *a_2_* < *b_1_*, *b_2_*.

Gnathosoma (Figures 2B; 3A, B; 4A): buccal structure comparable to that of *Saxidromus delamaraei* (Coineau & Naudo, 1986), *Bovidromus roussouwi* Coineau et al. 2006 and *Rhinodromus lootsi* Coineau et al. 2006 with four lips, with a particular disposition of the lateral lips with coaptation on ventral and paraxial zones and showing lamellar fringe expansions (“lacinulae”) (*la*) (Figure 3B).

Chelicerae (male and female) (Figures 2B; 3A, B, C; 4A, B, C) show lineate to ruminate microsculpture (Figs 3B, C), cheliceral body a large, broad hump, very similar in both sexes (Figure 4B, C). Positions and number of setae are very specific with some similarities and in other respects large differences: in common they have simple *cha* setae and bifid *chb*. Numbers of other setae differ significantly with only four in females; and males presenting a very rare form of neotrichy. This secondary multiplication of setae is a significant phenomenon, resulting in 16 to 20 setae. This bristle assembly forms a veritable brush, paraxially situated.

The denticulate, sickle-shaped inferior digit *mi*, situated opposite the superior digit *ms*, is an important primitive character indicated by Grandjean 1949, Coineau 1974 and Coineau et al. 2006. Blade *n* on paraxial surface of digit *im* situated on the distal zone of the inferior margin. Blade *n*, the *la* and the positions of *mi* and *ms*, may play an important role in feeding.

Transversal furrow establishing the posterior limit of sclerite ***D*.** The posterior dorsal part is composed of one undivided sclerite ***P*.**

Sclerite ***P*** (Figures 1A, 2A), presenting three pairs of depressed areas; seven pairs of setae (*c_1_*, *c_2_*, *d_1_*, *d_2_*, *e_1_*, *e_2_*, *e_3_*), lyrifissures *im* and *ip* and two tendon attachments. Depressed area 9 clearly visible in SEM and hardly observable by LM.

**Female.** Dorsal region differs greatly from that of the male. The female displaying two well defined sclerites ***A*** and ***M*** (Fig. 6A, 7A), separated by two transverse furrows. One at level of posterior zone of leg IV and another at the level of the space between epimeres III and IV. A third sclerite ***P*** (Fig. 6A, 7A) situated behind the second transversal furrow; considerable variation was observed in this sclerite. Only two examples of the most extreme variations are illustrated: Figure 7A with only one undivided sclerite ***P*** with anterolateral incisions, and Figure 8A with sclerite ***P*** divided into four paired and one unpaired microsclerites. Several variations between the two extremes were observed, sclerites are more or less visible but asymmetric variations were never found.

Sclerite ***A*:** unpaired structure, triangular to polyhedral; setae *pa* situated anteriorly to *bo*; depressed area 1 (unpaired) situated in sagittal plane, behind depressed area 2 (paired); this last area situated in front of and close to setae *po*. The *oc*, *op*, *ly*, the microsculpture and depressed area 3 (paired) is similar to male. In the posterior zone and near the first transversal furrow, we observed setae *a_1_*, *a_2_*, and the 4 paired depressed areas (Figure 8A).

Paired sclerite ***M***, ovoid to rectangularly shaped, situated between first and second transverse furrows: *ia* situated in anterior antiaxial angle of the sclerite; two depressed areas (5, 6) aligned longitudinally, and setae *b_1_* and *b_2_*.

Sclerite *P* (Figures 7A; 8A) can be observed with either of two characteristics: 1) unique unpaired sclerite (Figure 6A) presenting shape, structure, setal disposition (*c_1_*, *c_2_*, *d_1_*, *d_2_*, *e_1_*, *e_2_*, *e_3_*) and depressed areas (7, 8, 9) similar to male (Figure 1A). 2). Paired ovoid to polyhedral microsclerite presenting setae *c_1_*, *c_2_*, depressed areas 7 and 8, and lyrifissure *im*, surrounded by striate microsculpture (Figure 8A), and another paired microsclerite with setae *d_1_*, *d_2_*. Finally there is an unpaired, crescent-shaped sclerite (Figures 8A, 10A, B), clearly discernible in dorsal and posterior views with setae *e_1_*, *e_2_*, *e_3_* and depressed area 9.

The posterior view permits a clear comparison of the last dorsal sclerite of the two sexes. In the male (Figure 10B) the absence of the dorsal microsclerite containing *e_1_*, *e_2_*, *e_3_* setae is clearly visible; the opposite is found in the female (Figure 10A), where the crescent-shaped microsclerite is perfectly observable. Other ventral sclerites such as ***PS*** and ***AD*** and the anal opening are clearly visible in both cases.

#### Ventral region

(Figure 1B). **Male.** Sternal region bearing setae (*st*) with epimeric formula (3-2-3-3). Aggenital region occupied on either side by a large aggenital sclerite (***AG***), semicircular, with 3 pairs of aggenital setae. Progenital lips surrounding large genital opening, with five pairs of setae. An aligned row of 16 pairs of short setae occurs along paraxial border.

Anal segment surrounding anal opening (***AN***), with four pairs of setae; adanal segment (***AD***) outwardly more or less bean-shaped with four pairs of adanal setae; more paraxially ***PS*** segment with five pairs of setae.

Lyrifissure *ih* clearly visible, slightly closer to the anterior margin of *PS* sclerite

**Female** (Figure 7B; 10C). Shape of sternal region resembling that of male; epimeric formulae (3-3-2-3). Aggenital region occupied by large ***AG*** sclerite, more or less triangularly shaped, with three pairs of simple setae.

Progenital lips surrounding genital opening with 5-7 pairs of simple setae. Numerous simple setae aligned along paraxial edge. Due to the short distance between the progenital lips and the paraxial edge, and the almost equal lengths of setae, it is difficult to determine the exact number of both types of setae.

Other segments ***AN***, ***AD*** and ***PS***, and the number and disposition of setae are similar to that of male.

#### Legs.

**Male.** The legs present characteristics of other South African species (Coineau et al. 2006). Cuticular microsculpture on tibiae and tarsi in the form of linear striae (Figs 6A, B, D), while for other segments (trochanter, femur, genu) microsculpture is principally a polygonnetwork (Figure 6C), but in the basal zone cuticular striae are always present. Apotele in all legs, with three heteromorphic claws with a pair isomorphics (*ol*), and a small unpaired medial hook (*oc*) (Figures 5B, C; 6A, B). Two different types of barbs are presented by the paired claws (indicated in Figures 6A, B, with simple and double arrow): triangularly shaped barbs with a tooth-like appearance of relative length (length 5–7 µm, width in basal zone 1.5–2 µm) (Figure 6A, B, simple arrow); while the other type is a thin curved barbs (indicated Figure 6A, double arrow).

Leg I of Saxidromidae males show secondary sexual characters related to their role during the mating ritual, when lifting the female. They are generally more developed than that of females. In all four genera tarsus and tibia I constitute a pincer-like structure with grabbing function.

In *Saxidromus* the conversion to a pincer affects relative movement of the setae, and a depressed soft integumental area allows tethering of legs II and IV of the female close to their base (Coineau 1976, Figure N, page 239). Amongst males of these three South-African genera the locking of female legs is enhanced by the existence of hypertrophied setae.

*Nannodromus* and *Bovidromus* show a hypertrophied seta on tarsus I, and as in the other genera *Nannodromus* shows a hypertrophied seta (*ot*) at the tip of a true spur (*erg*) (French: ergot) at the tip of the tibia.

*Rhinodromus* exhibits a curved integumental thickening at the level of the upper part of the tibia near the tibiotarsal articulation (Coineau et al. 2006, Fig. 11C).

Several differences in sexual dimorphisms are indicated (Coineau et al. 2006); of which one is the hypertrophied setae. In *Nannodromus reveilleti* these are found on the tibia and tarsi (in Figs indicated as black setae) of the first pairs of legs (Figures 5B, C). Tibia I presenting a tibial claw (*ot*) situated on a protuberance (*erg*) (Figs 6D, E, F).

**Female.** Characteristics of legs similar to those of male, but principal sexual modifications are found in legs IV, not on the first pair as in males.

**Figure 11.**
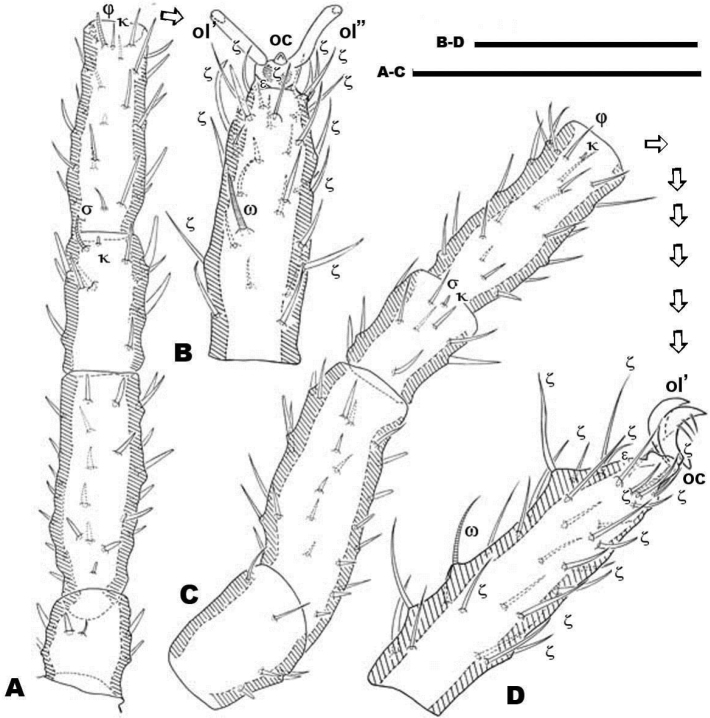
*Nannodromus reveilleti* gen. n., sp. n. Female. Legs I **A**, **B** dorsal view **C**, **D** lateral view. Scale bar: **A, B** = 100 μm; **C–D** = 50 μm.

Pairs I, II, III (only first pair illustrated, Figures 11A, B, C, D) presenting three heteromorphic claws as do males, also with the presence of two types of barbs (Figure 10E) (see above). Legs IV bearing three isomorphic claws.

**Figure 12.**
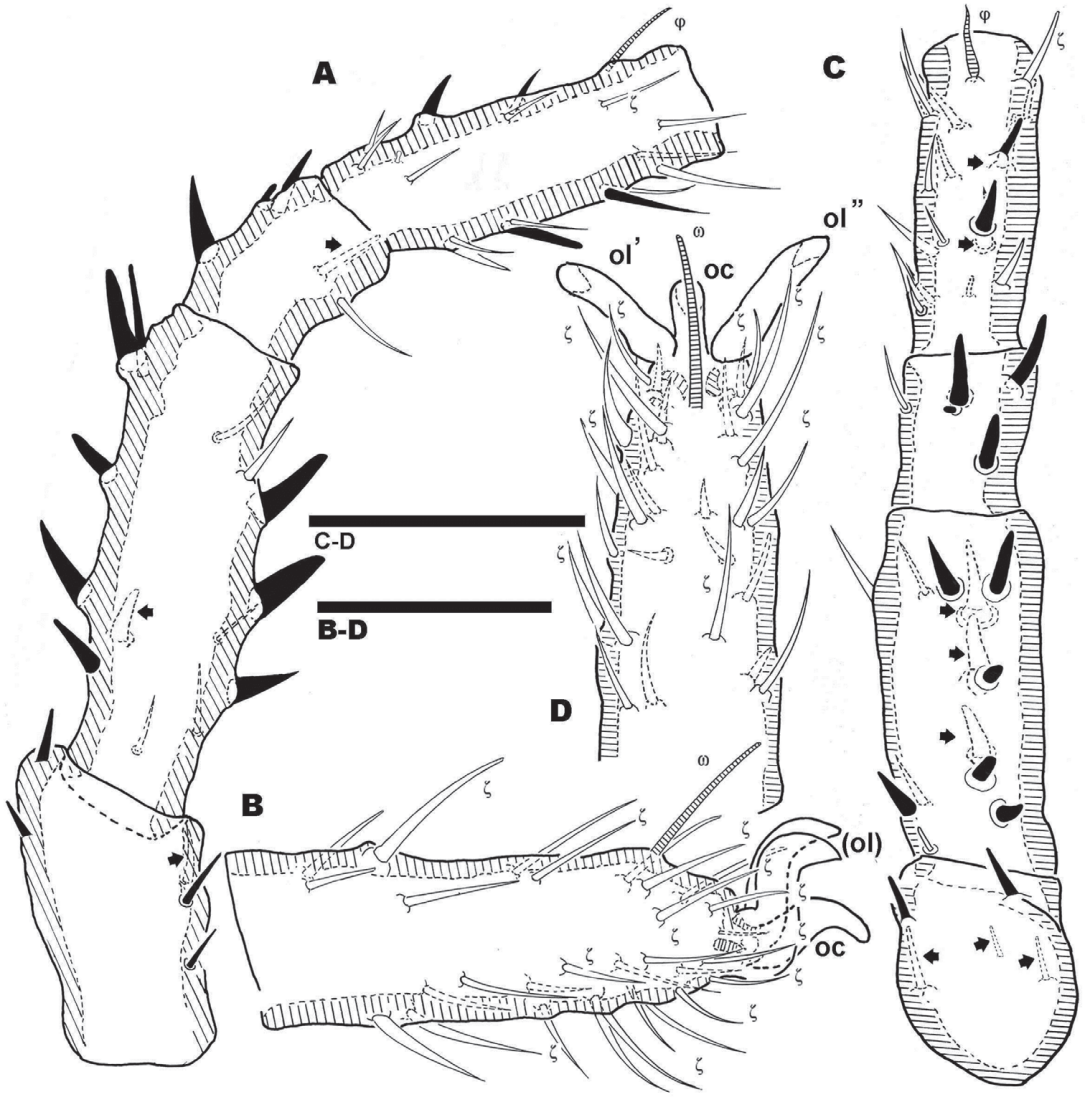
*Nannodromus reveilleti* gen. n. sp. n. Female Legs IV **A**, **B** dorsal view **C**, **D** lateral view. Scale bar: **A**, **B** = 70 μm; **C–D** = 40 μm.

Large number of hypertrophic setae (Figures 12A, C) (indicated as black setae or with black arrow depending on the side of the leg on which they are found) on leg pairs IV (Figures 12A–D).

#### Remarks.

This present study of legs is provisional as we are at the moment conducting further detailed leg studies of the four known genera.

The large number of setae, and the presence among them of euphathidia, solenidia, *k* setae and hypertrophic setae necessitated meticulous study with large enough quantities of material, as well as highlighting the need for the study of immature stages. Figure 10D permits observation of the size and shape of the claw, but several setae as well as their positions are lost. Our intention with this figure is to show the real shape of the claws.

## Discussion

This is divided into two parts, the first a comparison of the three South-African genera (*Bovidromus*, *Rhinodromus* and *Nannodromus*) and the second a comparison and discussion of feeding in *Saxidromus*.

### Comparison of the three South-African genera

The three genera present a series of similarities and differences:

**Size.** Important differences in size: *Bovidromus* (±900 × 450 µm); *Rhinodromus* (±845 × 380 µm) and *Nannodromus* (±610 × 215 µm); thus *Bovidromus* > *Rhinodromus* > *Nannodromus*.

**Dorsal structure.** Both sexes of *Bovidromus* exhibit a unique transverse furrow delimiting two dorsal sclerites, ***D*** and ***P*.** In *Rhinodromus*, the male and female exhibit two transverse furrows delimiting three dorsal sclerites (***A***, ***M***, ***P***).

*Nannodromus* presents, in the male, a dorsal structure with only one transverse furrow and two dorsal sclerites (***D***, ***P***); while in females at least two transverse furrows delimit three sclerites ***A***, ***M***, ***P***; sclerite ***P*** in some instances divided into several microsclerites. Without considering the detail of the subdivision of sclerite ***P***, it can be stated that the genera *Bovidromus* and *Rhinodromus* display a similar dorsal structure for both sexes, while in *Nannodromus* the male displays one type of dorsal structure and the female another.

**Setae e_2_ and e_3_.** Present in all three genera.

**Sexual dimorphism. Male.** *Bovidromus* 1) Large paired dorsal process, horn-shaped, arching upwards, situated apically on the aspidosoma. 2) One pair of triangular ridges, with tips facing each other at level of trichobothria and situated in paraxial position. 3) One pair of outward directing lateral horns, situated at level of trichobothria in antiaxial position.

Legs. Apotele of all legs of similar type with three heteromorphic claws consisting of a pair of isomorphics and a small unpaired medial hook. Leg I: tibia presents a tibial claw situated on a protuberance; hypertrophic setae found on femur, genu, tibia and tarsi; larger number on tibia and less on tarsi.

*Rhinodromus* 1) Large, unpaired cylindric process, curving slightly upwards, situated apically on aspidosoma. 2) One pair of horns, each directing outwards, situated in front and slightly paraxial to trichobothrium.

Legs. Apotele as in *Bovidromus*. Leg I: tibia with tibial claw situated on protuberance; hypertrophic setae on tibia in smaller numbers.

*Nannodromus* 1) Small, paired digitiform dorsal process, arching slightly upwards, situated apically on aspidosoma.

Legs. Apotele as in *Bovidromus*. Leg I: tibia with tibial claw situated on a protuberance; hypertrophic setae on tibia and tarsus.

**Females.** Legs. Apotele I-III, similar type in *Bovidromus*, *Rhinodromus* and *Nannodromus*, with three heteromorphic claws consisting of a pair of isomorphics and a small unpaired medial hook. Sexual dimorphism is found in leg IV, presenting three large claws and a series of hypertrophied setae along the leg.

Leg IV of *Nannodromus reveilleti* displaying hypertrophic setae on trochanter, femur, genu, tibia and tarsus. Hypertrophic setae absent from leg I.

**Chelicerae.** Characters in common: cheliceral body large, broad, and hump-shaped; setae *cha* simple; *chb* bifid; similar in both sexes.

Characters which differ: other setae: In all cases there is high neotrichy in males and low neotrichy in females: *Bovidromus* male ± 40 setae; female ± 12 setae; *Rhinodromus* male ± 16 setae; female ± 3 setae; *Nannodromus* male ± 20; female ± 4.

**Feeding in *Saxidromus*.** First observation of the chelicerae, with mobile falciform digit with teeth opposite fixed digit, and massive dentition may suggest the equipment of a predator (Lindquist 1979) such as *Labidostoma*; but more detailed study of the chelicerae (Coineau and Naudo 1986), supplemented with field observations (Coineau and Kovoor 1982; Coineau et al. 2006), several examinations of the digestive system (personal observation) and ultrastructural studies (Alberti 2012 – personal communication) all confirm that the Saxidromidae are microphytophages.

The chelicerae show a series of very important details such as: 1) the mobile digit exhibits an elevated blade throughout the middle distal zone of inferior edge; this structure probably contributing towards collection of particles from rock surface. 2) The system of lamellae (lacinulae) of lateral lips, which by brushing retain the charge of particles at the entrance of mouth, which have the chaelicerae. 3) The form in which the digits and body of chelicera are situated and connected (clearly observed in lateral view) shows significant suppleness, to maintain contact with substrate while absorbing the irregularities and retaining effectiveness for tiny particle collection.

The digestive system, in many dissections, shows the presence of particles, while ultrastructural studies show that the anterior pharynx extends deeply into the idiosoma and the esophagus presents a very wide lumen.

**Field observation.** In large numbers of field observations and during filming, mites often stopped and scraped vigorously on the rock surface possibly to obtain food.

## Supplementary Material

XML Treatment for Nannodromus

XML Treatment for Nannodromus reveilleti
